# Filling in the Blanks: Senior Medical Student Supporting the Transition of Incoming First-Year UK Medical Students During COVID-19

**DOI:** 10.1007/s40670-021-01361-4

**Published:** 2021-08-17

**Authors:** Ellen Lois Nelson-Rowe

**Affiliations:** Centre for Medical Education, School of Medicine, Cardiff University, Cochrane Building, Heath Park, Cardiff, CF14 4XN United Kingdom

**Keywords:** Transition, Medical school, Online resources, Near-peer support

## Abstract

There is a lack of resources available to support transition into the first year of UK medical schools. Due to COVID-19 and the possibility that students have lost learning, it is argued that there is a demand for free, accessible curated materials. These can reinforce confidence in expected core topics, mitigate differences in student knowledge and provide a head start in new concepts prior to entry — particularly important in the transition to a virtual learning environment. During lockdowns, this may lessen the effect of lost learning and can be assembled by medical students pitching appropriate content and encouraging near-peer support.

## Background

The impact of COVID-19 on medical education has resulted in a rapid adaptation into virtual learning for medical students [1], to ensure a continuation of learning. In the UK, unlike the USA, most entrants into medicine are around 18 years old (90%, with only around 10% being postgraduate) with the prerequisites being high grades in three advanced level exams (‘A levels’ — these are roughly equivalent to US SAT [Scholastic Assessment Test] and AP [Advanced Placement Exam]). This is in addition to admission tests: either the UCAT [University Clinical Aptitude Test] or BMAT [BioMedical Admissions Test] (like the MCAT [Medical College Admissions Test] in the USA). However, in 2020, incoming UK medical students (so-called offer holders) faced a disruption to their studies: a result of exam cancellations. The repercussions of this could ultimately affect the transition of the medical students into first year of the course — a transition widely recognised to be stressful [2], as students face a change in learning styles, increase in workload and new content together with all the lifestyle changes coincident with moving to university [3].

There is a lack of evidence of resources being made available and targeted for incoming first-year medical students to guide their preparation. Some medical schools that do provide reading lists in advance suggest broad and general resources [4], but it is not clear if students attempt or can easily access these for free ahead of their start. There appears to be a need to provide accessible, focussed academic resources to incoming students ahead of entering medical school [5]. In the USA, there have been numerous reports of prematriculation programs being offered for medicine — for example, Schneid et al. [6] reported an intensive 7-week face-to-face summer prematriculation program for ‘academically disadvantaged students’ [6]. Online resources have been described by Stoddard et al. [7] who developed an orientation program called ‘Fast Start’ to help incoming students particularly with anatomy [7], whilst Wilson et al. [8] released their didactic materials available to pre-matriculants [8]. All reported positive feedback as well as in most, improved performance once on the courses.

Such material may help to manage their expectations of the medical school curriculum [5, 9], which could perhaps increase confidence upon entry. This is particularly true for students affected by the pandemic who have suffered ‘lost-learning’ and not had the opportunity to revise for and sit high-stakes exams prior to entry. A new approach is described that was devised to support incoming medical students based on the hypothesis that they had experienced lost learning during the COVID-19 pandemic and involved the curation of online resources. These were pitched at an appropriate level to encourage students to revise and develop knowledge building upon their prior learning, with the introduction of material normally delivered in the first semester of medical course and thus ‘fill in the blanks’.

## Activity

Prior to developing resources, a scoping search of the literature was performed on the U.S. National Library of Medicine’s MEDLINE, Clarivate’s Web of Science and Elsevier’s Scopus databases to see if similar approaches had been previously taken. Key search terms used included the following: ‘medical student’, ‘medical school’, ‘transition’, ‘school pupil’, ‘sixth form student’ and ‘prematriculation’. The grey literature [10] was also searched for curated resources.

**Fig. 1:**
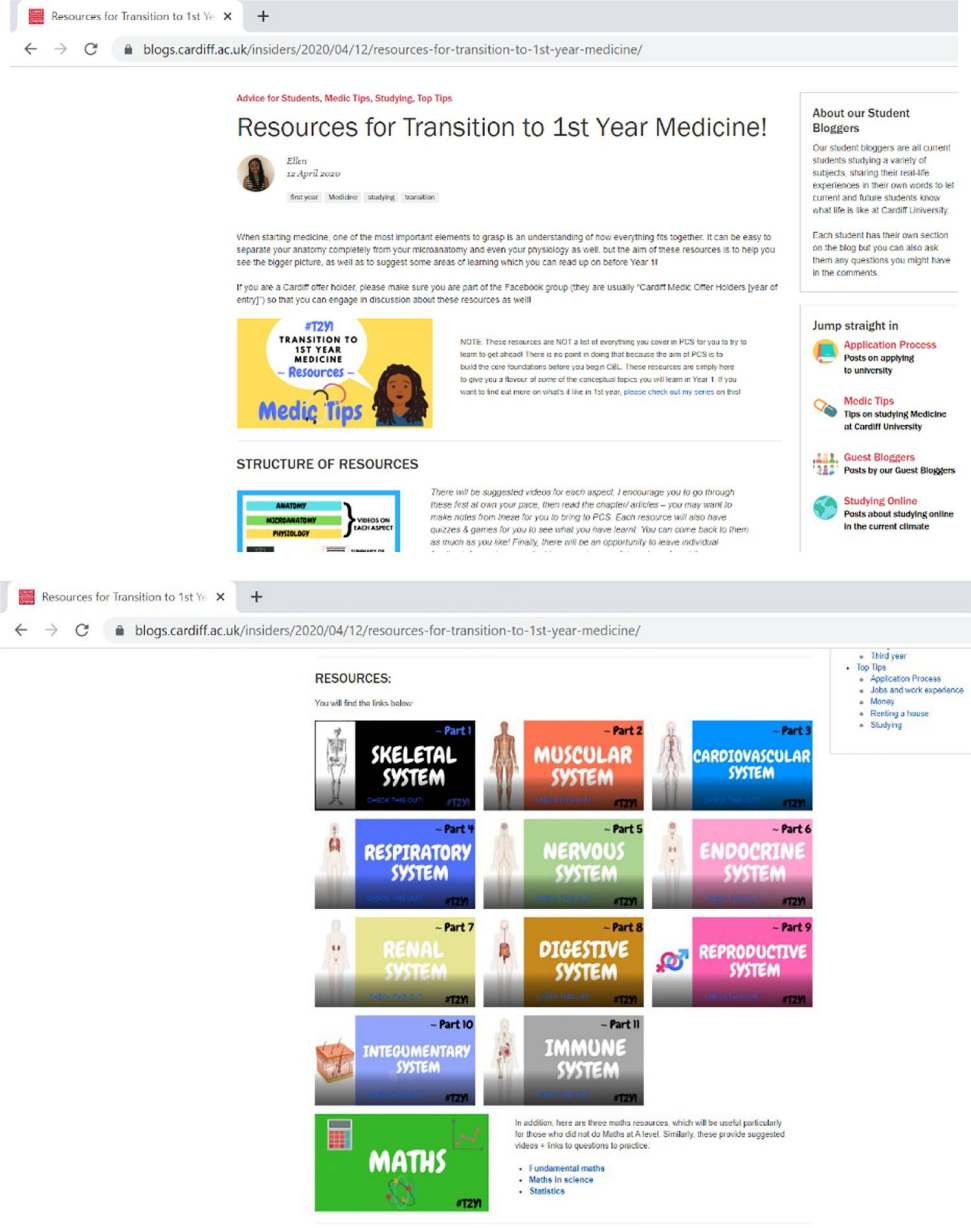
Illustrative screenshots of welcome page (top) and page showing the eleven main resources available as downloadable pdf files (bottom)

Based on each human body system, eleven main resources were created and were downloadable as a pdf file (Fig. 1). Each had learning outcomes to guide the focus of learning and was organised into subsections of anatomy, microanatomy and physiology, curated with a range of videos, articles/e-textbooks and quizzes. Most videos were duplicated to have two different sources explaining the same concepts. The resources were released weekly over a 3-month period so students who were aware of the resources could follow them weekly to have a task to do in their own time; alternatively, some students waited for all resources to be released to access later closer to starting medical school. Additionally, a set of collated maths resources based on three themes of fundamental maths, maths in science and statistics was released, to support students who did not do higher level maths. These resources were collated onto a Cardiff University blog page, with some suggested guidance of going through the videos first, then reading the relevant chapter or articles with the option to make notes if students wanted to bring them to their first semester and finally testing their knowledge using the quizzes and games on each resource. Students were encouraged to return to the resources as many times as they needed and were provided with an optional feedback form for each of the body system resources. The form contained three Likert-scale question items and two free text questions to inform duration of completion and general comments.

## Results

Since the release of the resources, between 12 April and 1 October 2020, the blog page had 3534 unique page views, with 4887 pure page views indicating returning students. It also has a number one ranked Search Engine Optimization, indicating its innovation [11].

Overall, 45 feedback responses were received. On average, each resource took between 2 and 4 hours to complete, with some students opting to spread content out over a few days. All students strongly agreed that the material helped to supplement and reinforce their confidence in topics they had previously learnt about, as well as finding the resources easy to use with a nice layout. Some other individual comments included that ‘the learning outcomes helped me stay focused and make notes on some of the relevant information’; ‘The mixture of written and visual resources helps to reinforce understanding and make useful notes’; and ‘I don't normally use videos as I find them difficult to make notes from — I think this will help me to practice’.

## Discussion

This initiative provided an opportunity for incoming medical students to engage with material relevant to their transition from secondary education to university over the first COVID-19 wave, at a time that they were experiencing ‘lost learning’ [12]. The high number of page views indicated wide interest beyond Cardiff medical school (around 670 offer-holders for an intake of around 300 students) and suggested a demand for this type of curated resource. Although available to any UK students accessing the blog, it was difficult to ascertain the number of individual link clicks on each resource (‘serious users’ as opposed to students viewing blog out of curiosity), due to limited blog data availability.

Even before the lost learning due to COVID-19, there is usually a gap between summer exams in the UK ending in July and entering medical school in September. This shows that the sustainability of such initiatives could be beneficial beyond COVID-19 and could be the basis of future research.

The uniqueness of the current study is that the resources were curated by a senior medical student (year 3, in consultation with faculty) and were used by predominantly 18-year-old students. The materials aimed to replicate the types of content suggested as pre-reading for tutorials in the first semester. It enabled students to try out popular sites such as *Khan Academy*, used by current medical students, to gain an insight into tried-and-tested resources they were likely to come across when they started their course.

The content included material suitable for the first semester science-intensive ‘Platform for Clinical Sciences’ (anatomy, physiology, biochemistry, cell biology, etc.) — part of the medical school’s curriculum as a prelude to the remaining part of the first two years all of which taught as case-based learning [13, 14] and relevant for most first-year medical courses in the UK. The curated material dealt with each body system (as years 1 and 2 focusses on basic and clinical science in the theme of the chronological life course). From a senior medical student perspective, materials were selected that built upon key topics normally revised during A-levels (roughly equivalent to SATs and APs), as well as including new concepts from the first semester of the medical course to provide the stimulus of new content. This may be viewed as a shortcoming, although selected by a student who had performed in the top quartile of their cohort in a year of 300.

The ‘old’ and ‘new’ were clearly indicated useful to iron-out disparities of content between syllabuses of examination boards that different students would have experienced. This was important as there is an assumption of an expected level of knowledge which is retained by students [15] so it gave them a chance to review concepts that they should understand before starting medical school. This also may help to reduce the knowledge retention gaps in the transition to university as concepts are refreshed, reducing the time needed to review concepts at the start of their medical course and focus on new content. Building upon a good core foundation to increase knowledge and developing the ability to learn is essential for full engagement with a medical course, a requirement of the regulatory body, The General Medical Council [16].

This also supported students transitioning from didactic methods of teaching to one of personal choice, regarding tackling learning concepts, as a way of developing self-regulated learning [17]. All resources being available online was essential: to help with students transitioning into a virtual learning environment and for accessibility.

Further research could assess the impact of the resources on student’s confidence having completed the first semester at medical school and perhaps assess the impact of provision of these resources on students who have been able to complete their A-level exams (*Deo volente*). Finally, the approach has highlighted the role that medical students can play in supporting incoming medical students as they have the best understanding of the appropriate contents that are best placed to ‘fill in the blanks’.

## References

[1] Rose S (2020). Medical student education in the time of COVID-19. JAMA.

[2] Simpson V, Halpin L, Chalmers K, Joynes V (2019). Exploring well-being: medical students and staff. Clin Teach.

[3] Briggs A, Clark J, Hall I (2012). Building bridges: understanding student transition to university. Qual High Educ.

[4] University of Oxford Medical Sciences Division. Introductory reading. 2019. https://www.medsci.ox.ac.uk/study/medicine/pre-clinical/applying/reading/. Accessed 18 Oct 2020.

[5] Kebaetse MB, Kebaetse M, Mokone GG, Nkomazana O, Mogodi M, Wright J (2018). Learning support interventions for year 1 medical students: a review of the literature. Med Educ.

[6] Schneid S, Apperson A, Laiken N, Mandel L, Kelly C, Brandl K (2018). A summer prematriculation program to help students succeed in medical school. Adv in Health Sci Educ.

[7] Stoddard H, Pamies R, Carver D, Todd G (2008). Developing an online prematriculation orientation program and its relation to student performance in the first class taken in medical school. Teach Learn Med.

[8] Wilson W, Henry M, Ewing G, Rehmann J, Canby C, Gray J (2011). A prematriculation intervention to improve the adjustment of students to medical school. Teach Learn Med.

[9] Bassett AM, Brosnan C, Southgate E, Lempp H (2018). Transitional journeys into, and through medical education for first-in-family (FiF) students: a qualitative interview study. BMC Med Educ.

[10] Adams J, Hillier-Brown FC, Moore HJ, Lake AA, Araujo-Soares V, White M (2016). Searching and synthesising ‘grey literature’ and ‘grey information’ in public health: critical reflections on three case studies. Syst Rev.

[11] Smith A. Tips on improving your company’s SEO ranking. 2020. https://channels.theinnovationenterprise.com/articles/how-can-i-improve-my-seo-ranking/. Accessed 29 Nov 2020.

[12] Dhawan S (2020). Online learning: a panacea in the time of COVID-19 crisis. J Educ Technol Syst.

[13] Rimmer A. Undergraduate course aims to “attract, train, and retain” doctors in Wales. BMJ. 2014. 10.1136/bmj.g2093

[14] Cardiff University. School of Medicine undergraduate degree programmes entry 2022. 2021. https://www.cardiff.ac.uk/__data/assets/pdf_file/0004/1325821/MedicineUG_2022_web-13_05_21_Final.pdf/. Accessed 6 July 2021.

[15] Jones H, Black B, Green J, Langton P, Rutherford S, Scott J (2015). Indications of knowledge retention in the transition to higher education. J Biol Educ.

[16] Council GMCaMS. Achieving good medical practice: guidance for medical students. 2016. https://www.gmc-uk.org/-/media/documents/achieving-good-medical-practice-20200729_pdf-66086678.pdf/. Accessed 18 Oct 2020.

[17] Sandars J (2010). Pause 2 Learn: developing self-regulated learning. Med Educ.

